# A descriptive analysis of PVL-positive multidrug-resistant Staphylococcus aureus in hospital-associated infections in Saudi Arabia

**DOI:** 10.6026/97320630016586

**Published:** 2020-08-31

**Authors:** Waleed Mazi, Fawaz Alshammari, Jun Yu, Md Jahoor Alam, Mohd Saeed, Khalid Alshaghdali, Amir Saeed

**Affiliations:** 1 Department of Clinical Laboratory Sciences College of Applied Medical Sciences, University of Hail-Hail-Kingdom of Saudi Arabia; 2 Infection Prevention and Control Department, King Abdul Aziz Specialist Hospital-Taif, Saudi Arabia; 3 Strathclyde Institute of Pharmacy and Biomedical Science, University of Strathclyde, Glasgow, United Kingdom; 4 Karolinska Institute, Department of Laboratory Medicine, Division of Clinical Microbiology, Karolinska University Hospital, Huddinge, SE-141 86 Stockholm, Sweden; 5 Department of Biology, College of Science, University of Hail, Hail-Kingdom of Saudi Arabia

**Keywords:** Methicillin resistant Staphylococcus aureus, hospital and community- associated MRSA infections

## Abstract

Methicillin resistant Staphylococcus aureus infections impose a huge risk to public health in healthcare and community settings worldwide. Therefore, it is of interest to document data on the anti-biogramas and genotypes of isolates from Saudi Arabia. We assessed the antimicrobial susceptibility, determined spa (protein A gene) and analyzed multilocus MLST genotypes, and detected PVL gene in these isolates. We collected 28 clinical MRSA isolates, cultured and determined the minimum inhibitory concentrations of 17 antimicrobial agents using Vitek2 system (BioMerieux, USA) from 3 hospitals in Saudi Arabia during the year 2012. Polymorphic region of the spa and seven housekeeping genes were amplified and sequenced. BioNumerics v.5.1 (Applied Maths) was used for spa typing and MLST. Samples were screened for the presence of PVL and mecA genes using polymerase chain reaction (PCR). Analysis shows that all isolates were susceptible to chloramphenicol, rifampicin, nitrofurantoin, teicoplanin, daptomycin and vancomycin. The T4573/ST22 strains are found to be prevalent in the Saudi Arabia (N=6, 21%). We further noted that three isolates (t363/ST240 strain) were resistant to eight antimicrobial agents. Most of t4573/ST22 strains were PVL positive, resistant to ciprofloxacin and linked to HA-MRSA infections. We document data for the presence of emerging multi drug resistant S. aureus strains carrying the PVL gene circulating within hospitals. This highlights the urgent need for continuous active surveillance and implementation of prevention measures.

## Background

Staphylococcus aureus infections are a major cause of community-acquired (CA-) and hospital-acquired (HA-) infections and are associated with significant morbidity and mortality rates. In 1999, National Healthcare Safety Network-USA (NHSN) reported that 52.3% of nosocomial infections occurring in ICUs were due to methicillin resistant Staphylococcus aureus (MRSA) [1]. HAIs caused by multidrug- resistance have become a serious public health problem, especially in intensive care units (ICUs) due to patients' existing condition, impaired immunity and exposure to many invasive devices and antibiotic treatments, leading to increasing cost, morbidity and mortality [2-4]. Infections with CA-MRSA in patients with no contact with the hospital environment have been described worldwide [5-8]. Exposure of CA-MRSA strains to the selective pressure of antibiotics used in hospitals has the potential for selection of expanded antibiotic resistance profiles in the pathogen. Consequently, constant re-introduction of MRSA into hospitals from the community reservoir could significantly hamper infection control efforts [7,9]. CA-MRSA strains are polyclonal, with an epidemiological association between clone type and geographical origin, whereas HA-MRSA infections are caused by a limited number of clones [7, 10,11]. The rate of MRSA infection ranges from 12% to 49%, with variations in susceptibility pattern profiles, but there are very limited data on the prevalence of different genotypes in Saudi Arabia [12-15]. A systemic review and meta-analysis of the prevalence of MRSA in the Kingdom of Saudi Arabia, based on 26 articles published between 2002 and 2012, revealed variation in the prevalence and antimicrobial susceptibility in patients, but also very limited data on genotyping and virulence gene detection [16]. Panton-valentine leukocidine-positive Staphylococcus aureus causes recurring skin and soft tissue infections. Recently, a molecular characterization study on MRSA infections in Saudi Arabia showed a high diversity clonal complex encoding for Panton Valentine Leukocidine (PVL) and carrying resistance markers in CA-MRSA strains [17]. It is of interest to document data on the anti-biogramas and genotypes of multi drug resistance PLV gene positive S. aureus strains from Saudi Arabia.

## Methodology

### Bacterial isolates: 

Twenty-eight clinical MRSA isolates were collected randomly from three referral hospitals in Saudi. Isolates were cultured on sheep blood agar and incubated at 37°C for 24 h. S. aureus isolates were identified as round white colonies positive to Gram stain, catalase and slide agglutination tests [18].

### Detection of MRSA isolates:

Methicillin-resistant phenotype was confirmed according to British Society for Antimicrobial Chemotherapy (BSAC) standards using the Vitek2 system (BioMerieux, USA). An isolate is considered methicillin resistant when the minimum inhibitory concentration (MIC) breakpoint of oxacillin is >2 mg/L and of cefoxitin >4 mg/L [19].

### Antimicrobial susceptibility testing:

The MIC of 17 antimicrobial agents, namely; cefoxitin, chloramphenicol, ciprofloxacin, clindamycin, daptomycin, erythromycin, fusidic acid, gentamicin, linezolid, mupirocin, nitrofurantoin, oxacillin, penicillin, rifampicin, tetracycline, teicoplanin and vancomycin, was determined according to BSAC standards using the Vitek2 system (BioMerieux, USA). DNA extraction: Genomic DNA was isolated from a 2-mL overnight culture with the DNeasy tissue kit (QIAGEN, Hilden, Germany), using lysostaphin (100 mg/L; Sigma, Taufkirchen, Germany).

### Staphylococcal protein A (Spa) and Multi locus sequence typing (MLST-typing):

The polymorphic regions of the spa gene and seven housekeeping genes (arc, aroE, glp, gmk, pta, tpi and yqiL) were amplified by PCR using the primers (listed in Table 1), as described previously [20,21]. All sequencing reactions were carried out using the ABI PRISM BigDye Terminator cycle sequencing ready reaction kit (Applied Biosystems, Foster City, California, USA). The sequence data were analysed in BioNumerics v.5.1 (Applied Maths).

### Detection of PVL and mecA genes:

A PCR was carried out for PVL and mecA gene amplification individually in two runs using a thermo cycler (Eppendorf, Hamburg, Germany) as described previously with modifications [22]. In brief, 20 µL of final reaction mixture of each run containing 10 µL of 1X Taq master-mix reaction, 1 µL of each primer (100µM) (listed in Table 1) and 2 µL of the DNA (20 ng) template and mecA-F: 5'- mecA-R: 5'- were used. PCR conditions were as follows: initial denaturation at 95°C for 10 min, followed by 25 cycles of denaturation at 95°C for 1 min, annealing at 74°C for 1 min and extension at 72°C for 1 min and a final extension step at 72°C for 10 min. MRSA ATCC 35591 and DNase/RNase-free distilled water were included as positive and negative controls, respectively. For visualisation of the product, 10 µL of PCR amplicons were mixed with 1 µL of EzVision One loading dye (Amresco, Solon, OH, USA) and loaded into a 1.5% (wt/vol) agarose gel (Agarose I™). Electrophoresis was carried out in 1X TAE buffer at 80v for 50 minutes. A molecular weight marker 100- bp ladder (Promega, Madison, WI, USA) was included on each gel. Bands were visualised using an Alpha Innotech UV imager (FluorChem™). MRSA infections: Identification of HA-MRSA and CA-MRSA infections was based on hospitalisation history and site of infection according to CDC/HNSN criteria [23]. An isolate was defined as HA-MRSA if the MRSA-positive specimen was obtained 2 days after hospital admission and met CDC site infection criteria. An isolate was defined as CA-MRSA if the MRSA-positive specimen was obtained within 48 hours of admission, with no history of MRSA infection within a year as a risk of acquired MRSA.

## Results

All MRSA isolates proved to be susceptible to chloramphenicol, daptomycin, nitrofurantoin, teicoplanin, rifampicin and vancomycin. However, resistance rates varied as follows: fusidic acid (46%), tetracycline (39%), ciprofloxacin (36%), trimethoprim (28%), gentamicin (25%), clindamycin (21%), erythromycin (14%) and mupirocin (7%). Three strains demonstrated resistance to eight antimicrobial agents (ciprofloxacin, clindamycin, erythromycin, fusidic acid, gentamicin, tetracycline, trimethoprim and mupirocin). Twelve spa types were identified, including t4573 (21%) followed by t304 (18%) and t044, t267 and t363 (10% of each). Eight multi locus sequence types (MLSTs) were identified; with the majority being sequence type (ST) ST-22 (32%), followed by ST-80 and ST-97 (14%), ST-2882 (11%), ST-241 (7%) and ST-239, ST-5 and ST-6 (3%). The t4573/ST-22 genotype was most prevalent in the hospital (46%). All isolates tested positive for mecA gene. Thirteen MRSA isolates (46%) tested positive for PVL gene; 54% related to HA-MRSA infection and 46% to CA-MRSA infection. All t4573/ST22 strains were resistant to ciprofloxacin, harboured PVL gene and were related to surgical site infections, skin soft tissue infections and pneumonia (Table 2).

## Discussion

MRSA infections are prevalent in healthcare and community settings and more prevention efforts are needed to overcome newly emerging multidrug resistance. The incidence rates and susceptibility patterns to antimicrobial agents are variable across geographical regions, due to many factors such as surveillance methods, economic status and use of antimicrobials. Here, we have conducted an investigation on MRSA strains from Taif hospitals in Saudi Arabia. MRSA isolates were collected randomly January-August 2012 from three governmental hospitals serving 1400000 populations in Taif and Hail city; King Abdul Aziz Specialist Hospital (KAASH), King Khalid Hospital (KFH) and Children Hospital (CH) to assess HA-MRSA and CA-MRSA infections including antimicrobial susceptibility pattern, MLST and spa-typing, PVL gene detection. All MRSA isolates were susceptible to chloramphenicol, daptomycin, nitrofurantoin, rifampicin, teicoplanin and vancomycin. There is wide variation in the antimicrobial susceptibility pattern in Saudi Arabia. Rifampicin resistance has previously been observed in Al Khubar (76%), Qassem (60%), Taif and Riyadh (33%), Aseer (11% and Makkah (6%), but not in Najran city [24-30]. Chloramphenicol resistance has been recorded in Taif (30%) and Asser (1.5%) [25-26]. Only one case of daptomycin resistance has been reported in Hail, Saudi Arabia, in Najran (9.4%) [28]. This variation can be assumed to be a reflection of differences in study design and surveillance method. Multidrug-resistant S. aureus has been reported in many hospitals world-wide but, to the best of my knowledge, this thesis is the first study to report multi-drug resistant MRSA with resistance to ciprofloxacin, clindamycin, erythromycin, fusidic acid, gentamicin, mupirocin, tetracycline and trimethoprim in Saudi Arabia.

A diversity of spa and MLST types were identified in Hail, with t4573/ST-22 being the most prevalent in the hospital (21%). Similar diversity has been reported in Riyadh [30,31]. Interestingly, although t4573, harbouring PVL gene and resistant to ciprofloxacin, is common in Taif city (n=6, 21%), the strain has a very rare distribution worldwide and is not well characterised. One isolate has been reported in Saudi Arabia (Riyadh) [30], one in New Zealand [32] and two in Sweden (www.spaserver.ridom.de). One isolate of t037 was detected in Taif, but t037 is common in Riyadh (35%) and has a wide global distribution. This variation probably reflects the low sample collection in the present study. Clonal complex (CC) 22 is the most common MLST type (N= 9, 32%) followed by CC97, CC80, CC241, CC2882, CC6, CC5 and CC239 in Taif-Saudi Arabia. Similarly, Monecke and colleagues identified the same CCs, in addition to CC1, CC9, CC30, CC45 and CC88 in Riyadh- Saudi Arabia [17].

PVL-positive MRSA strains are associated with complicated infections, especially skin infections and necrotising pneumonia. High prevalence of the gene encoding PVL was noted in Taif city (46%) and tended to be more associated with HA-MRSA infection (62%) compared with CA-MRSA (39%). In contrast, Monecke et al. demonstrated high prevalence of PVL-positive (54%) samples in strains considered CA-MRSA in Riyadh [17]. This variation is most likely the result of the data and samples being obtained from a hospital-based survey and not a population-based study. All ST80 strains detected here tested positive for PVL gene. Moreover, all ST80 strains tested positive for PVL gene. Similarly, PVL positivity has been detected in MRSA ST 80 genotype in many countries [33-39]. Although Bin Nejam and colleagues demonstrated that all MRSA ST80 Tunisian isolates were intermediately susceptible to fusidic acid [33], all of our MRSA ST-80 strains were resistant to fusidic acid. This variation probably reflects differences in antibiotic use between the countries. Fucidic acid is commonly used for local treatment and is one of the antimicrobial agents used for elimination of MRSA in hospitals in Saudi Arabia. The proportion of HA-MRSA infections (50%) was higher than that of CA-MRSA infections (25%) in this study. Similarly, Eed EM et al. observed in a recent study that HA-MRSA infections were more common than CA- MRSA infections in Taif [25]. However, the higher prevalence of HA-MRSA compared with CA-MRSA in this study probably does not reflect the true values, because the data and samples were obtained from a hospital-based survey and not a population-based study.

No correlation was found between positivity to PVL and CA/HA-MRSA infections. CA-MRSA has several molecular characteristics compared with HA-MRSA, for example SCCmec types V and IV usually produce PVL and are generally susceptible to non-β-lactam antibiotics [7,40]. However, time-based definition of CA-MRSA with the recently changing epidemiology of MRSA infections may make it difficult to differentiate between CA-MRSA and HA-MRSA infections [7]. PVL-positive samples were observed in both CA-MRSA and HA-MRSA categories. Similarly, a previous study demonstrated that global CA-MRSA outbreaks could occur in the presence or absence of PVL gene [7]. It is possible that CA-MRSA category infections (SCCmecA types I, II or III) are transferred to, and colonise, patients or hospital environments and may cause hospital infections, thus becoming HA-MRSA category. We state limitations on the epidemiology of MRSA infections in Taif city due to (1) lack of SCCmecA-typing; (2) small sample size; and (3) dataset is through hospital-based surveillance.

Association between MRSA ST-80 and PVL positive is found without susceptibility to antimicrobial agents. An in-depth study using a larger number of clinical MRSA ST-80 samples is required to establish such association. Thus, we document data for the presence of emerging multi drug resistant S. aureus strains carrying the PVL gene circulating within hospitals in Saudi Arabia. This highlights the urgent need for continuous active surveillance and implementation of prevention measures.

## Conclusion

The presence of multi drug resistance PLV gene positive S. aureus strains circulating within hospitals in Saudi Arabia warrants the need for continuous active surveillance and implementation of prevention measures.

## Declaration on Publication Ethics:

The authors state that they adhere with COPE guidelines on publishing ethics as described elsewhere at https://publicationethics.org/. The authors also undertake that they are not associated with any other third party (governmental or non-governmental agencies) linking with any form of unethical issues connecting to this publication. The authors also declare that they are not withholding any information that is misleading to the publisher in regard to this article.

The authors are responsible for the content of this article. The Editorial and the publisher has taken reasonable steps to check the content of the article with reference to publishing ethics with adequate peer reviews deposited at PUBLONS.

## Figures and Tables

**Table 1:** List of primers:

Gene	Forward 5'-3'	Reverse 5'-3'
Spa	AGACGATCCTTCGGTGAGC	GCTTTTGCAATGTCATTTACTG
Arc	TTGATTCACCAGCGCGTATTGTC	AGGTATCTGCTTCAATCAGCG
aroE	ATCGGAAATCCTATTTCACATTC	GGTGTTGTATTAATAACGATATC
Glp	CTAGGAACTGCAATCTTAATCC	TGGTAAAATCGCATGTCCAATTC
Gmk	ATCGTTTTATCGGGACCATC	TCATTAACTACAACGTAATCGTA
Pta	GTTAAAATCGTATTACCTGAAGG	GACCCTTTTGTTGAAAAGCTTAA
Tpi	TCGTTCATTCTGAACGTCGTGAA	TTTGCACCTTCTAACAATTGTAC
Yqi	CAGCATACAGGACACCTATTGGC	CGTTGAGGAATCGATACTGGAAC
PVL	ATCATTAGGTAAAATGTCTGGACATGATCCA	GCATCAAGTGTATTGGATAGCAAAAGC
mecA	GTAGAAATGACTGAACGTCCGATAA	CCAATTCCACATTGTTTCGGTCTAA
Spa gene primers are specific for spa genotyping. Arc, aroE, Gip, Gmk, Pta, Tpi and Yqi genes primers are specific MLSA typing. MecA gene primers are specific for identification of MRSA. Luk gene primers are specific for identification of PLV strain.

**Table 2:** Genotypes and susceptibility pattern of MRSA infections observed at KAASH, Taif-Saudi Arabia, 2012.

Isolate	Spa-type	MLST	CC	PVL	Site	Type of infection	Resistant to
A11	t304	2882		Negative	SST	CAI	-
A34					Nasal	Colonization	
A3		6	45		Blood	HAI	
A4				Positive	SST	CAI	
A21		2882		Negative	Nasal	Colonization	Intermediate susceptible to linezolid and vancomycin
F21	t311	5	45				Cli
F2	t2770	97			Blood	HAI	Fa
F7				Positive	SST		Fa
A2	t044	80	80		BURN		Fa
A7	t4573	22	22	Negative	SST	CAI	Cip
F14/F15				Positive		HAI	Cip
A23					SSI		Cip
A30							Cip, Tr
F16	t748				SST	CAI	Cip, Tr
A32	t5716			Negative	Nasal	Colonization	Cli, Tr
F23	t223						Tr, Te
F13	t044	80	80	Positive	SST	HAI	Fa, Te
F20						CAI	Fa, Te
F9	t131						Fa, Te
A5	t037	239	8				Fa, Te
A29/A13	t267	97		Negative	Nasal	Colonization	Fa, Te, Gan
A28					SSI	HAI	Fa, Te, Gen
C3	t4573	22	22	Positive	PNEU	HAI	Cli, Cip, Tr, Gen, Ery
A15/A22	t363	241	8	Negative	BURN		Cli, Fa, Cip, Tr, Te, Gen, Ery, Mu
A27							Cli, Fa, Cip, Tr, Te, Gen, Ery,
A: King Abdul Aziz Specialist Hospital, F: King Faisal Hospital, C: Children Hospital C, CC: clonal complex, HAI: hospital acquired infection, CA: community acquired infection, SST: skin soft tissue, Cli: clindamycin, Fa: fucidic acid, Cip: ciprofloxacin, Tr: trimethobrium, Te: tetracycline, Gen: gentamicin, Ery: erythromycin, Mu: muperocin, Clo: chloramphenicol, Ref: refampicin. The table describes the genotypes and distribution of MRSA infections. There is a diversity of CC distribution in the study. Majority of strain type t4573, MLST 22, CC22 harboured PVL gene with different in susceptibility pattern to antimicrobial agents. However strain t363, MLST 241, CC8 doesn't harbour PVL but resistant to many antimicrobial agents.
